# Role of Microstructures in the Dielectric Properties of PVDF-Based Nanocomposites Containing High-Permittivity Fillers for Energy Storage

**DOI:** 10.1021/acsami.2c23013

**Published:** 2023-03-02

**Authors:** Leontin Padurariu, Elisabetta Brunengo, Giovanna Canu, Lavinia Petronela Curecheriu, Lucia Conzatti, Maria Teresa Buscaglia, Paola Stagnaro, Liliana Mitoseriu, Vincenzo Buscaglia

**Affiliations:** † Faculty of Physics, Alexandru Ioan Cuza University, Blv. Carol I, nr.11, 700506 Iasi, Romania; ‡ Department of Chemistry and Industrial Chemistry, University of Genoa, Via Dodecaneso 31, 16146 Genoa, Italy; § CNR-SCITEC, Institute of Chemical Sciences and Technologies “Giulio Natta”, National Research Council, Via de Marini 6, 16149 Genoa, Italy; ∥ CNR-ICMATE, Institute of Condensed Matter Chemistry and Technologies for Energy, National Research Council, Via de Marini 6, 16149 Genoa, Italy

**Keywords:** polymer matrix composites, 3D FEM modeling, energy storage, ferroelectrics, electric properties

## Abstract

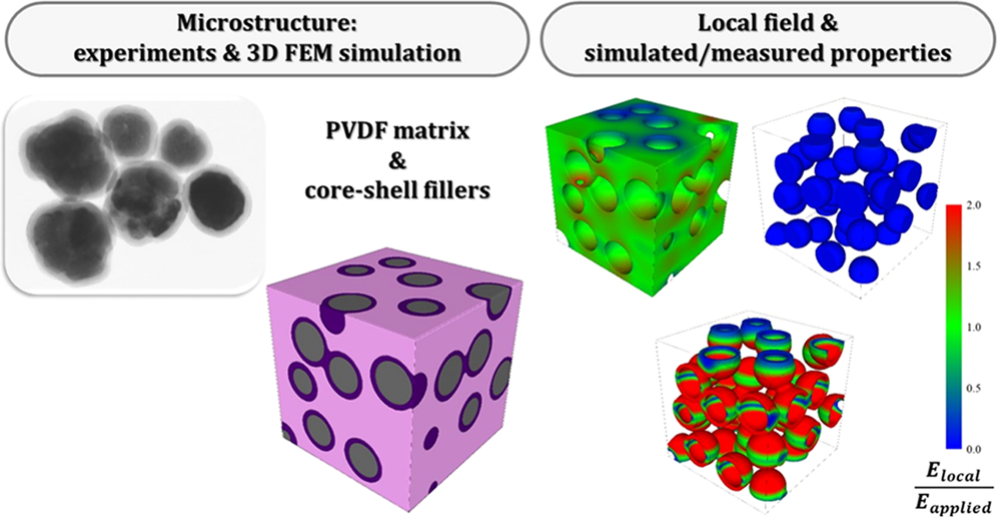

Polymer-based nanocomposites containing inorganic ferroelectric inclusions, typically ABO_3_ perovskites, have emerged as innovative dielectric materials for energy storage and electric insulation, potentially coupling the high breakdown strength (BDS) and easy processing of polymers with the enhancement of dielectric constant provided by the ferroelectric phase. In this paper, experimental data and three-dimensional finite element method (3D FEM) simulations were combined to shed some light on the effect of microstructures on the dielectric properties of poly(vinylidene fluoride) (PVDF)-BaTiO_3_ composites. The existence of particle aggregates or touching particles has a strong effect on the effective dielectric constant and determines an increase of the local field in the neck region of the ferroelectric phase with a detrimental effect on the BDS. The distribution of the field and the effective permittivity are very sensitive to the specific microstructure considered. The degradation of the BDS can be overcome by coating the ferroelectric particles with a thin shell of an insulating oxide with a low dielectric constant, such as SiO_2_ (ε_r_ = 4). The local field is highly concentrated on the shell, while the field in the ferroelectric phase is reduced almost to zero and that on the matrix is close to the applied one. The electric field in the matrix becomes less homogeneous with increasing the dielectric constant of the shell material, as happens with TiO_2_ (ε_r_ = 30). These results provide a solid background to explain the enhanced dielectric properties and the superior BDS of composites containing core–shell inclusions.

## Introduction

Polymer-based composites containing insulating inorganic fillers are attracting a great deal of interest as potential dielectric materials for high-energy-density capacitors and other applications, including embedded planar capacitors, electroluminescent displays, and insulating layers. They potentially combine typical properties of polymers, such as easy processing at low temperature, flexibility, and high breakdown strength (BDS) with the high relative dielectric constant (or dielectric permittivity, ε_r_′) of some inorganic compounds and, in particular, of ferroelectric perovskites such as BaTiO_3_, (Ba,Sr)TiO_3_, and Pb(Zr,Ti)O_3_. These perovskites have ε_r_′ of the order of 1–5 × 10^3^ and loss tangent (tan δ) of 0.01–0.02 at room temperature, though their BDS is much lower than that of polymers. Among polymer matrices, poly(vinylidene fluoride) (PVDF) and PVDF-based copolymers are largely investigated because of their relatively high dielectric constant (about 10), low dielectric losses (tan δ = 0.02–0.05), ferroelectric character, and a remarkable BDS of 1500–5000 kV/cm. Since the energy density in a capacitor is proportional to the permittivity of the dielectric, composites with high ε_r_′ are preferred in energy storage applications.^1−4^ However, the effective permittivity of the composite increases slowly with the volume fraction of inorganic filler, and substantial amounts of ceramic particles are needed to get a permittivity a few times higher than that of PVDF. For example, the addition of 30 vol % BaTiO_3_ particles leads to values of ε_r_′ in the range 20–40, depending on composite processing, filler synthesis method, surface functionalization of filler particles, as well as on microstructural features such as shape and size of the inclusions.^3^ In general, an increase of composite BDS can be attained by reducing the particle size to a few tens of nm, provided that a homogeneous dispersion of the fillers is realized.^5−7^ The shape and orientation of nonspherical particles (sheets and fibers) have a strong effect on dielectric properties.^8^ Composites containing nanofibers aligned perpendicular to the direction of the applied field (the most common case) show the minimum increase of dielectric response and the maximum improvement of BDS.^9^ Composites containing a limited amount of BaTiO_3_ nanofibers (either neat or coated) often show higher permittivity than the materials containing spherical particles, at least for low filler amounts.^3^ However, the use of spherical fillers is most common and simplifies the material processing.

Surface functionalization of BaTiO_3_ has been widely investigated as a strategy to enhance the matrix–filler compatibility and, thus, the adhesion of the polymer on the inorganic surface.^3,4,10−17^ Surface functionalization can facilitate the dispersion of the inclusions in the polymer matrix, leading to a more homogeneous microstructure. While suppression of the loss tangent is generally observed, the effect on the dielectric constant is contrasting.

A further strategy to improve the dielectric properties of PVDF-BaTiO_3_ composites is by coating the ferroelectric inclusions with a film of a highly insulating binary oxide, such as SiO_2_,^18−20^ Al_2_O_3_,^21−24^ TiO_2_,^25−29^ and MgO.^30^ Some common trends have been identified irrespective of the shell nature and filler morphology (equiaxed particles or fibers/wires): the BDS is increased while the losses and leakage current are reduced, resulting in the improvement of the energy charge/discharge efficiency despite a lowering of the dielectric constant is generally observed. Two qualitative explanations have been proposed to elucidate the beneficial effect. First, the highly insulating coating avoids the formation of conducting pathways between percolating inclusions.^3,31^ Second, the interposition of an additional layer minimizes the electric mismatch between matrix and ferroelectric inclusion (“buffer” layer concept).^3,4,14,26,30^ However, this latter concept has not been elaborated using quantitative models. In some cases, a relatively high dielectric constant (of the order of 50 for 30 vol % spherical inclusions) is observed, even for coated inclusions.^17,26^ A reason for the improved dielectric response could be related to the contribution of an additional dielectric relaxation process taking place around room temperature over a wide frequency range.

A further important factor that is neglected in most studies despite its potential impact on the dielectric properties of composites is that the filler particles (either neat or coated) often form aggregates rather than being isolated. Aggregates frequently originate at the synthesis stage and, especially in the case of nanoparticles, are retained in the ensuing composites. Looking at the fracture surface of many composites, the existence of particle aggregates seems quite common.^13,16,17,20,22,23,29,32^ Furthermore, when the amount of filler is significant (≥30 vol %), approaching the percolation threshold, particles come in contact for merely statistical reasons. Despite the large numbers of studies on polymer-based composites with inorganic fillers, a detailed understanding of the impact of the interparticle contacts and, more generally, of microstructure is still largely missing, while finite element modeling (FEM) usually does not take explicitly into account the existence of aggregates or touching inclusions.^16,17,21,25^ Cai et al.,^33^ in their notable article, have concluded, on the basis of 2D FEM simulations, that a nonuniform distribution of ceramic nanoparticles will aggravate the concentration of local electric field, thus slightly enhancing the dielectric response but seriously decreasing the BDS of nanocomposites. However, composites containing touching particles and coated inclusions were not considered, while a comparison with experimental data is missing.

In this paper, we combine 3D FEM calculations and experimental results to gain insight into the effect of particle aggregation and particle coating on the dielectric properties of PVDF-based composites containing spherical BaTiO_3_ inclusions.

Composites were prepared with neat BaTiO_3_ (BT) particles, TiO_2_-coated BaTiO_3_ (BT@TiO_2_) particles, and SiO_2_-coated BaTiO_3_ (BT@SiO_2_) particles. The binary oxides have been selected as inorganic coatings because they correspond to two well-distinct situations. While the dielectric constant of TiO_2_ is intermediate between PVDF and BaTiO_3_, the permittivity of SiO_2_ is lower than that of the other two components. Furthermore, both oxides are excellent insulators with a high BDS. The distribution of the electric field in the material and the effective permittivity have been calculated for composites containing 30 vol % inclusions with variable agglomeration degrees, either containing single-phase BT particles or core–shell structures (BT@TiO_2_ and BT@SiO_2_), and the results have been compared with the experimental data.

## Results and Discussion

### Microstructure and Phase Composition

**Figure 1:**
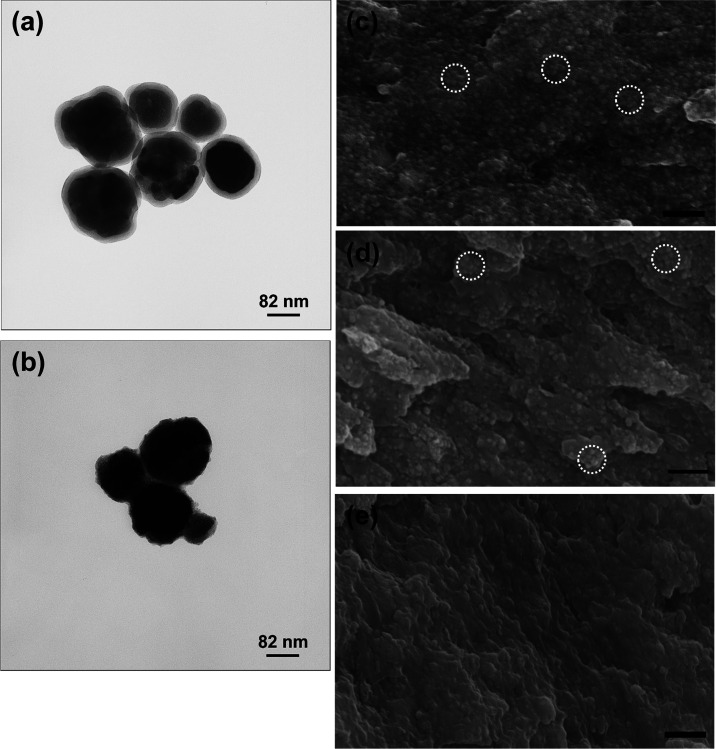
Morphology of (a) BaTiO_3_@SiO_2_ and (b) BaTiO_3_@TiO_2_ particles. SEM images of the fracture surfaces of (c) C-BT, (d) C-BT-S15, and (e) C-BT-T13 composites. The white circles show the location of some particle agglomerates. Bar: 1 μm.

A detailed microstructural characterization was performed on both the inclusions and the composites to evaluate the shell thickness and the filler distribution inside the polymer matrix. Representative images of the morphology of BT@SiO_2_ and BT@TiO_2_ particles are shown in Figure 1. The crystal structure of the BT nanoparticles (mean size of 125 nm) is pseudocubic, as indicated by the X-ray diffraction pattern reported in Figure S1. The silica coating is homogeneous with a nearly constant thickness, whereas the titania shell is more irregular. The estimated average shell thickness and relative shell volume are reported in Table 1. The composites are free of evident porosity and show a good dispersion of the inorganic inclusions though the formation of small aggregates is evident. For composites with neat BT and BT@SiO_2_ fillers, single particles and agglomerates are well visible, as shown on the composite fracture surface of Figure 1. For BT@TiO_2_ inclusions, only a limited number of isolated particles are observed, and most of the inclusions correspond to agglomerates with a size <1 μm.

**Table 1:** Shell Type, Average Shell Thickness, and Relative Shell Volume of the Different BaTiO_3_ Particles Used for the Preparation of the Investigated PVDF-Based Composites (Columns 2–4), Dielectric Constant, Loss Tangent, Recoverable Energy Density, and Efficiency of Composites (Columns 5–8)

composite	shell type	average shell thickness (nm)	volume fraction of shell (%)	dielectric constant at 1 kHz	loss tangent at 1 kHz	recoverable energy storage density (J·cm^–3^)	efficiency (%)
C-BT				31.8	0.030	0.287	52
C-BT-T6	TiO_2_	6	24.0	20.9	0.023	0.213	62
C-BT-T13		13	43.0	19.0	0.020	0.142	55
C-BT-T22		22	59.5	18.0	0.023	0.125	55
C-BT-S6	SiO_2_	5.5	22.4	17.8	0.020	0.129	38
C-BT-S15		15	47.5	13.9	0.024	0.098	41

According to the DSC measurements, the crystalline fraction in the polymer matrix is close to 50% in most samples and increases to ≈60% in composites with the BT@SiO_2_ inclusions (Table 2). The ATR-FTIR spectra (see Figure S2) indicate that the PVDF matrix contains only the polymorphs α and β with a predominance (up to 68%) of the latter phase in most composites (Table 2). The introduction of ceramic fillers significantly increases the fraction of ferroelectric β phase in comparison to the neat polymer, as already reported.^34^ The particles with the TiO_2_ shell seem especially effective, in agreement with previous work on composites with titania fillers.^35^

**Table 2:** Crystalline Fraction of PVDF (*X*_c_), Amount of β Phase Referred to the Crystalline Fraction (*F*_β_) and to Overall Polymer Amount (*F*_EA_) for the Neat PVDF Polymer, and the PVDF-Based Composite Films

composite	*X*_c_ (%)	*F*_β_ (%)	*F*_EA_ (%)
PVDF polymer	54	40	22
C-BT	50	57	27
C-BT-T6	51	60	31
C-BT-T13	49	64	31
C-BT-T22	49	68	33
C-BT-S6	61	53	32
C-BT-S15	59	48	28

### Low-Field Dielectric Properties

**Figure 2:**
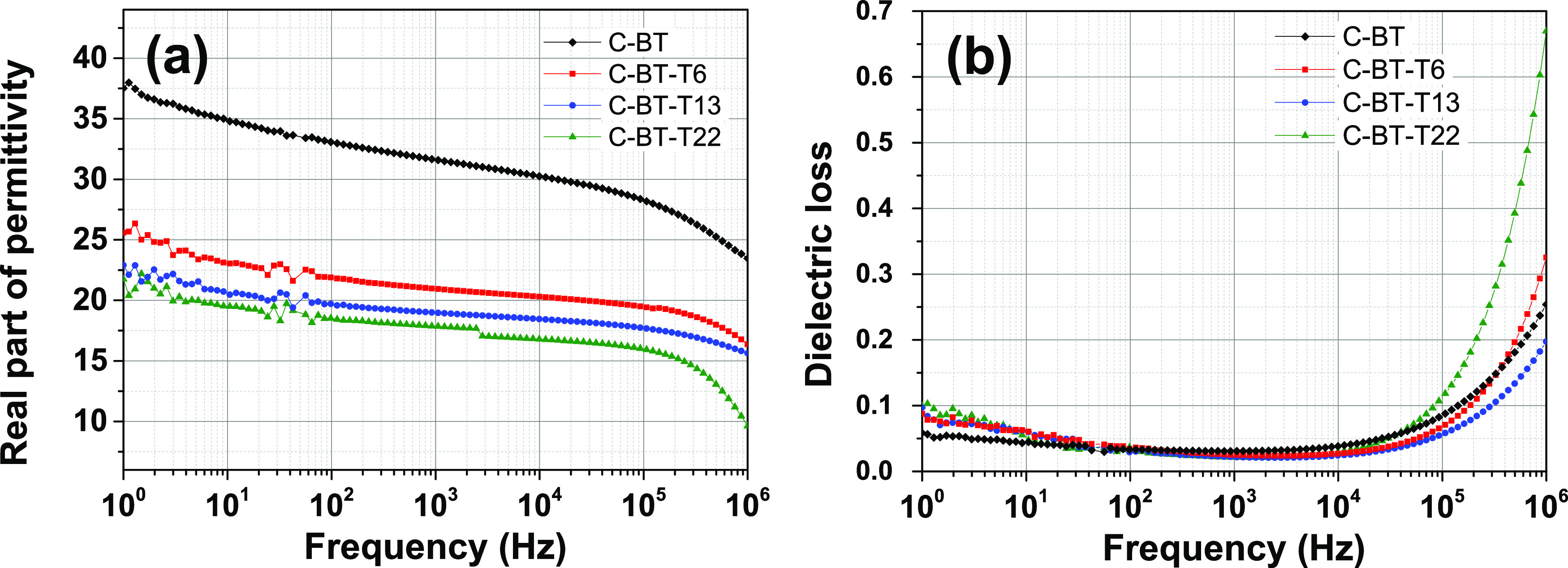
Room-temperature dielectric properties of PVDF composites containing 30 vol % of BT-Ty inclusions. (a) Relative dielectric constant. (b) Loss tangent. The data of composite C-BT are reported for comparison.

The low-field dielectric properties of C-BT-Ty composites are shown in Figure 2. The titania coating determines a systematic drop of permittivity with the shell thickness, from 32 (uncoated inclusions) to 21 (C-BT-T6), 19 (C-BT-T13), and 18 (C-BT-T22), respectively, at 1 kHz. For comparison sake, the permittivity of PVDF thick films processed with the same procedure is 10 at 1 kHz.^36^ The incorporation of coated particles does not determine an anomalous increase of permittivity at low frequency as observed in some composites and probably attributable to extrinsic interfacial polarization processes.^16,17,37^

**Figure 3:**
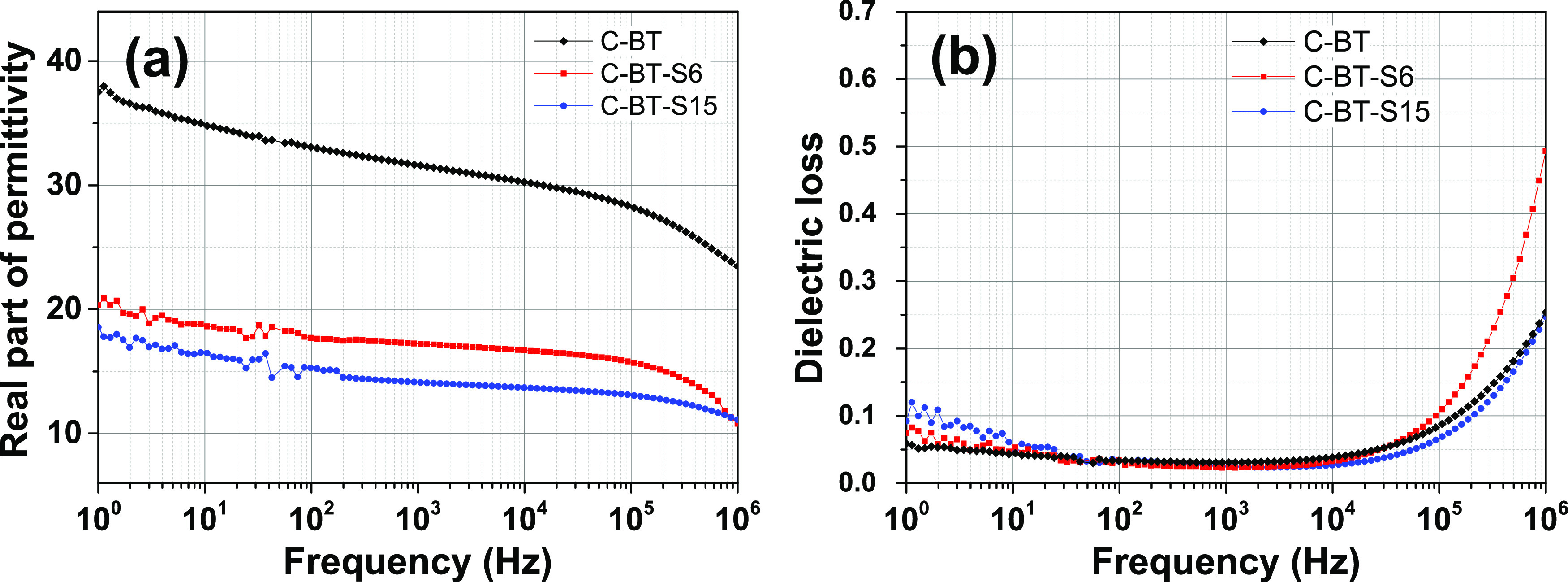
Room-temperature dielectric properties of PVDF composites containing 30 vol % of BT-Sy inclusions. (a) Relative dielectric constant. (b) Loss tangent. The data of composite C-BT are reported for comparison.

The presence of the TiO_2_ shell leads to a reduction of the dielectric losses to <0.03 in the range 10^2^–10^4^ Hz, the same level observed in the neat polymer.^38^ The loss tangent is increased at lower frequencies yet comparable to PVDF. Similar trends are also exhibited by composites containing the BaTiO_3_@SiO_2_ inclusions (Figure 3), though the permittivity undergoes a larger decrease. At 1 kHz, ε′ is 18 for C-BT-S6 and 14 for C-BT-S15. Irrespective of the coating nature, even a relatively thin shell of 6 nm (24% of the inclusion volume) is enough to suppress the dielectric constant by 34% (TiO_2_ shell) and 44% (SiO_2_ shell).

### High-Field Properties

**Figure 4:**
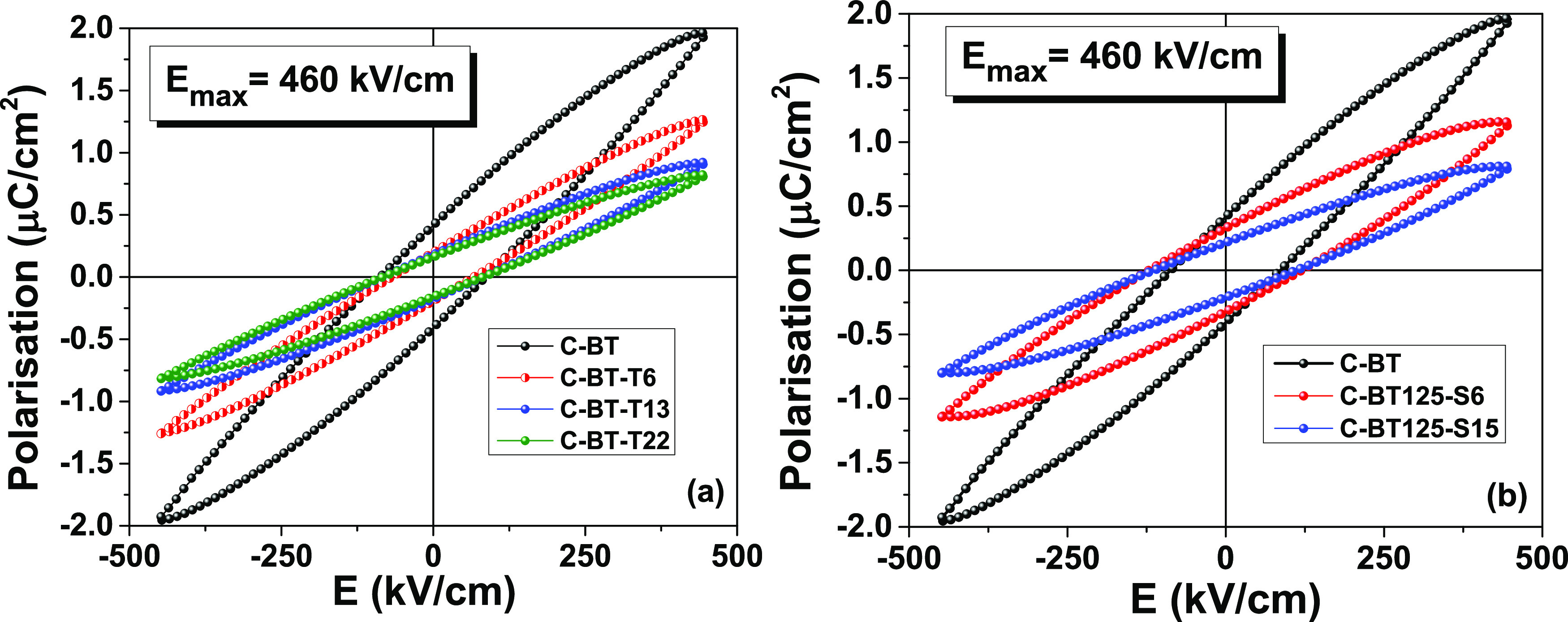
Dielectric displacement vs electric field loops at 10 Hz. (a) Composites with BaTiO_3_@TiO_2_ particles. (b) Composites with BaTiO_3_@SiO_2_ particles. The data of composite C-BT are reported for comparison.

The dielectric displacement vs field amplitude loops for the different composites measured at 10 Hz under a maximum field amplitude of 460 kV/cm are reported in Figure 4. In this field range, the nanocomposite materials behave as linear dielectrics with small losses. The maximum applied field is much lower than the coercive field of PVDF, and thus the dissipative motion and reorientation of the domains responsible for the polarization switching are not activated yet. Nevertheless, a significant impact of the filler coating can be observed. For all composites containing the core–shell particles, the loop area and, consequently, the losses are smaller than those of the material prepared using the uncoated particles. This indicates that the coating, either with TiO_2_ or SiO_2_, enhances the insulating properties of the composites, even at relatively high fields. Because of the lower losses, the C-BT-Ty composites have higher efficiency in energy storage (Table 1), although the energy density is inferior. The slant of the *P*(*E*) loops (Figure 4) decreases with increasing the shell thickness. This is a consequence of the diminishing effective permittivity, as the dielectric constant is proportional to the first derivative of the dielectric displacement with respect to the field.

### FEM Simulations

**Figure 5:**
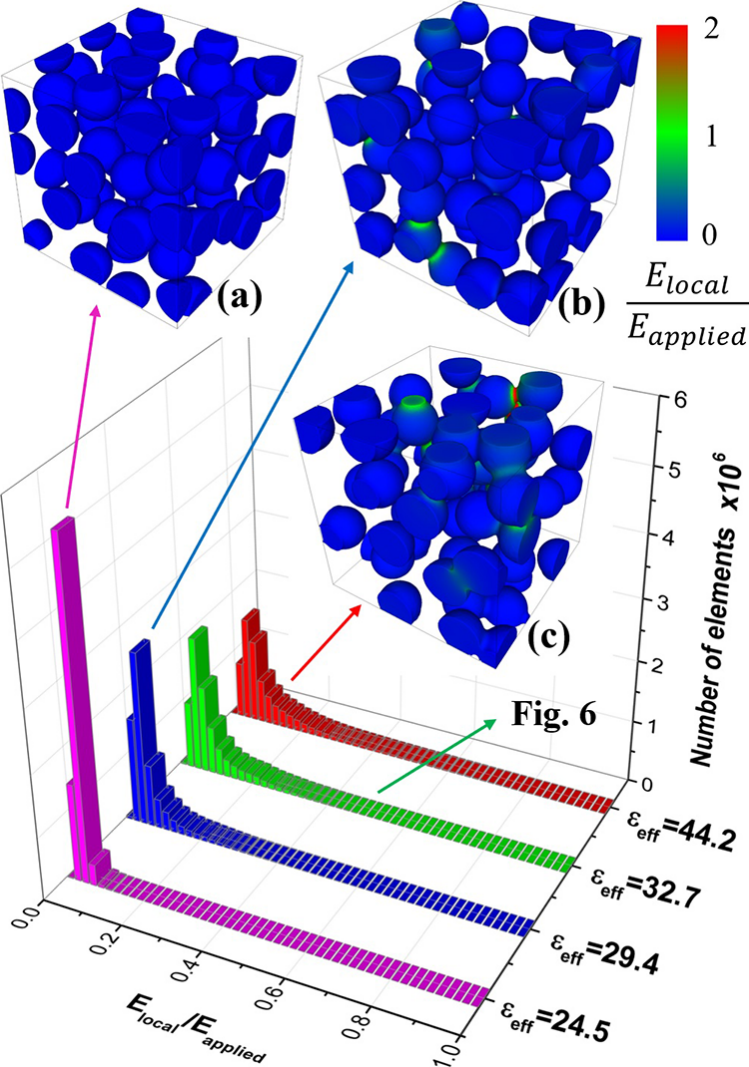
Local electric field maps and distributions in BT phase (30 vol %) as determined by FEM simulations in four PVDF-BT composites with different microstructures: perfectly isolated particles and ε_eff_ = 24.5 (a), touching particles and ε_eff_ = 29.4 (b), touching particles and ε_eff_ = 44.2 (c). The microstructure and local field maps for the composite with ε_eff_ = 32.7 are shown in Figure 6.

**Figure 6:**
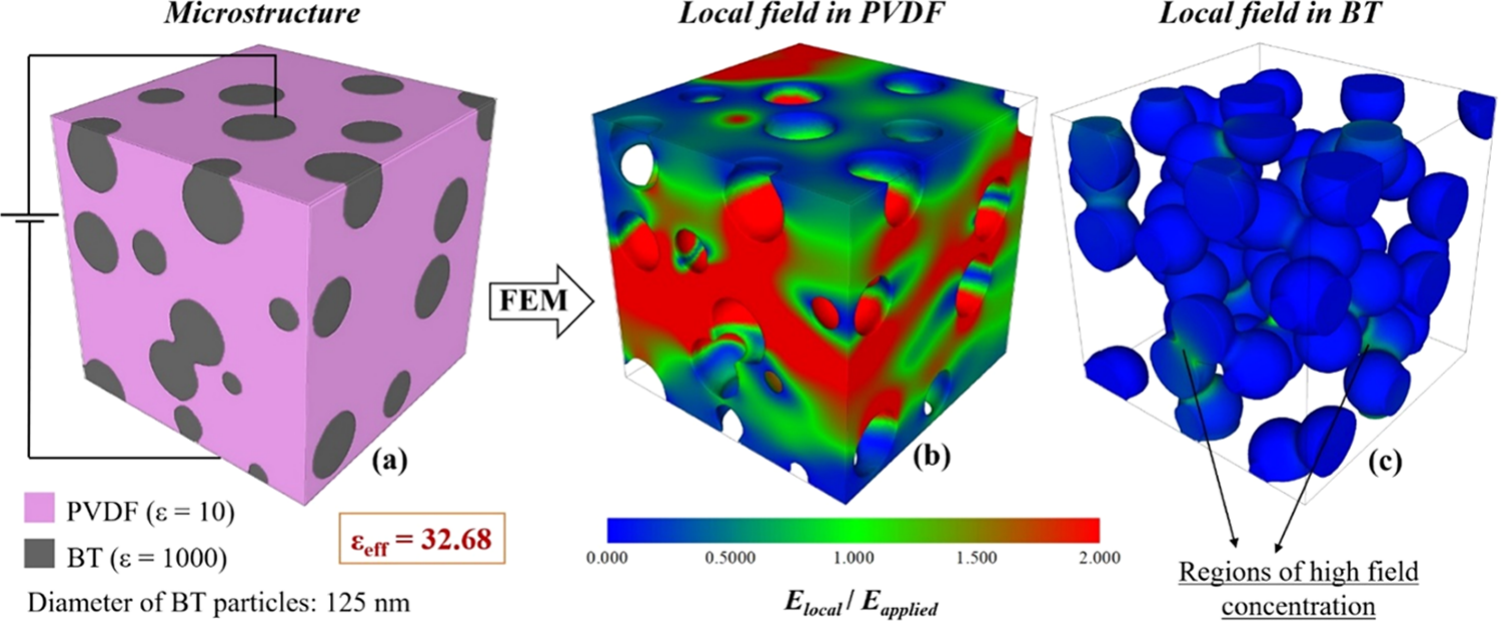
Microstructure of a (a) PVDF-BT composite containing 30 vol % BT particles with ε_eff_ = 32.7 and local field maps in (b) PVDF matrix and (c) BT inclusions simulated by FEM.

Some selected results of FEM simulations for composites containing uncoated BT particles are shown in Figure 5. The figure reports the normalized local field (*E*_local_/*E*_applied_) distribution in the BT phase of four distinct microstructures selected from a number of simulations. It is worth remembering that the microstructures were randomly generated each time. The first one (Figure 5a), corresponding to perfectly isolated particles, gives the lowest permittivity value of 24.5. For noninteracting inclusions, the effective permittivity is practically independent on the specific configuration considered. All three other microstructures, containing a different number of contact points located in different positions, give higher ε_eff_ values, up to 44.2. The existence of interparticle contacts has a substantial effect on the effective permittivity, which increases considerably in comparison to the ideal case of perfectly isolated particles. The average value resulting from several calculations is ≈32, close to the experimental result (Table 1). In the absence of contact points, the electric field in the BT inclusions is practically suppressed by the presence of the low permittivity matrix, which tends to concentrate in its volume the field lines (i.e., the lower permittivity dielectric is subjected to a higher field). The distribution of the electric field in the matrix is highly inhomogeneous irrespective of the existence of interparticle contacts. As an example, the distribution of the electric field in the matrix and BT inclusions for the composite with ε_eff_ = 32.68 is shown Figure 6. As discussed in a previous paper,^39^ the increase of ε_eff_ in ideal composites with respect to the pure polymer is related only to the enhancement of the local field in the polymer matrix and not to the contribution of the high-permittivity component to the total energy of the system. However, when some particles touch each other, the electric field in the contact region increases considerably and can get similar (green regions) or even higher (yellow and red regions in Figures 5c and 6c) values than the applied field. The enhancement of the local field in BT provides an additional contribution to the effective dielectric constant of the composite and points out the significant effect exerted by the existence of agglomerates or touching particles, i.e., by the microstructure. As shown in Figure 5, the electric field distribution in the inclusions is much broader than in the ideal case of isolated particles. In particular, the local field is maximized when the segment connecting the centers of the two interacting particles is perpendicular to the applied field (see the red region for case (c) in Figure 5). Consequently, the field on the filler can exceed the BDS of BT (100–150 kV/cm^40,41^), even when the applied field is far from the critical threshold. The local failure can then trigger the propagation of a fatal crack. The risk of local failure is less serious for the matrix because the BDS of PVDF is usually 1–2 orders of magnitude (1500–5000 kV/cm^3^) higher than that of the inclusions.

**Figure 7:**
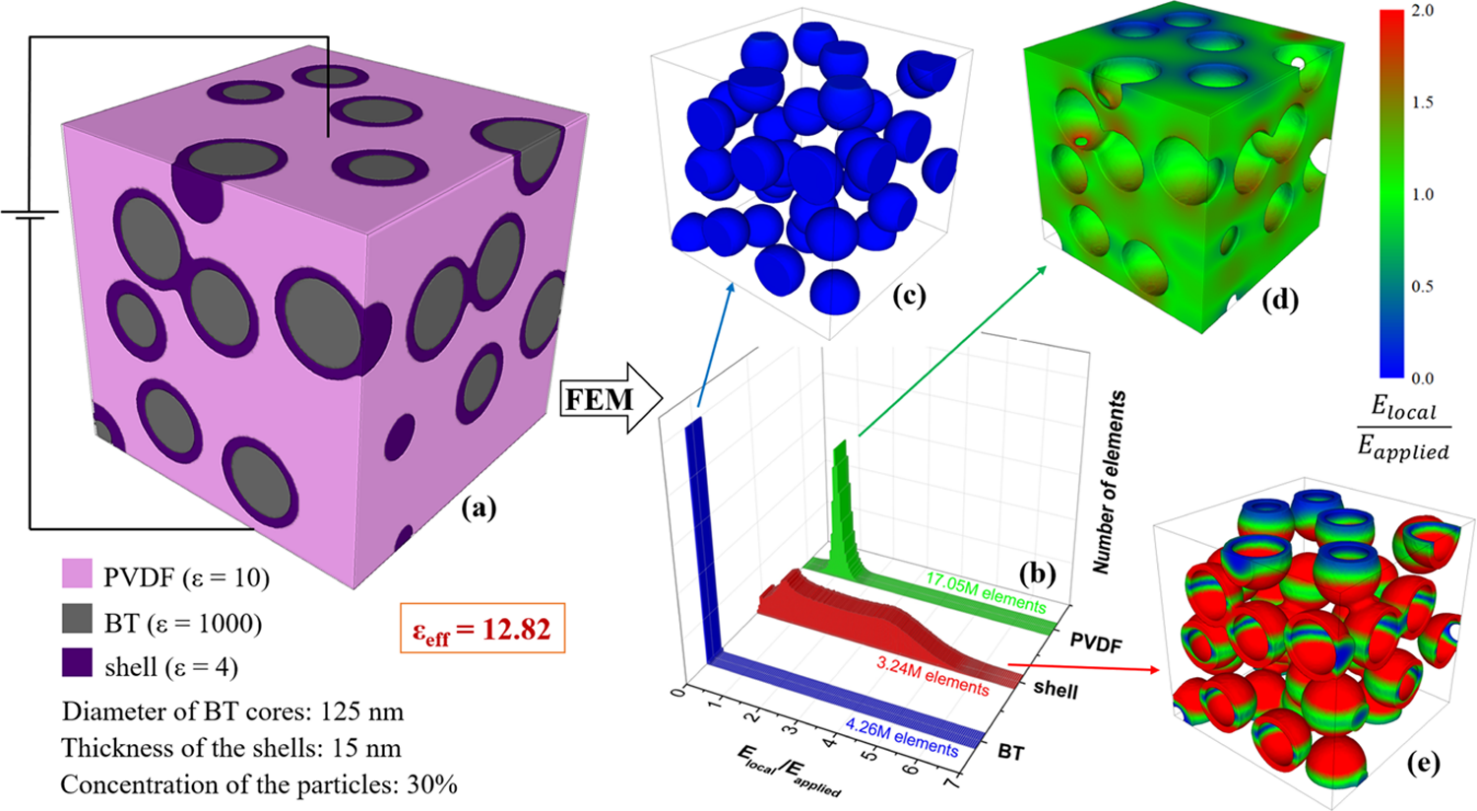
(a) Microstructure of a PVDF composite with BaTiO_3_@SiO_2_ inclusions (30 vol %), (b) local field distribution expressed on a number of elements basis and the local field maps (c) in the BT inclusions, (d) polymer matrix, and (e) silica shell, simulated by FEM. Shell thickness is 15 nm.

The presence of the SiO_2_ shell (ε_r_′ = 4) on the BT particles leads to a completely different electric field distribution (Figure 7). Even for touching inclusions, the electric field on the BT phase is virtually zero. The electric field on the matrix is relatively homogeneous and close to the applied field. In contrast, the field in the silica shell is highly inhomogeneous and strongly enhanced in comparison to the applied field, as apparent from the very broad local field distribution, which extends to values up to 6–7 times the applied field. Despite this strong increase of the local field, the BDS of silica is very high (5–15 × 10^3^ kV/cm^42^), and thus the composite can withstand very high applied fields. The BDS of PVDF composites with silica-coated BT inclusions is reported to be significantly larger than that of PVDF.^20^ Thus, the effect of the silica coating is to protect both the BT filler and the polymer matrix from breakdown. A further consequence of the homogeneous fields on both the BT particles and the matrix is that ε_eff_ is only weakly dependent on the specific microstructure. The main drawback of using a silica coating is the resulting lower effective permittivity of such composites, which is predicted to be 12.3 for isolated inclusions and 12.8 for touching particles for a shell thickness of 15 nm, values not far from the experimental result of 13.9. A strongly suppressed permittivity results in a drop of the energy storage density (Table 1). However, these drawbacks can be partly relieved using thinner coatings. The simulations indicate that when the shell thickness is 5 nm, the effective permittivity increases to ≈18.

**Figure 8:**
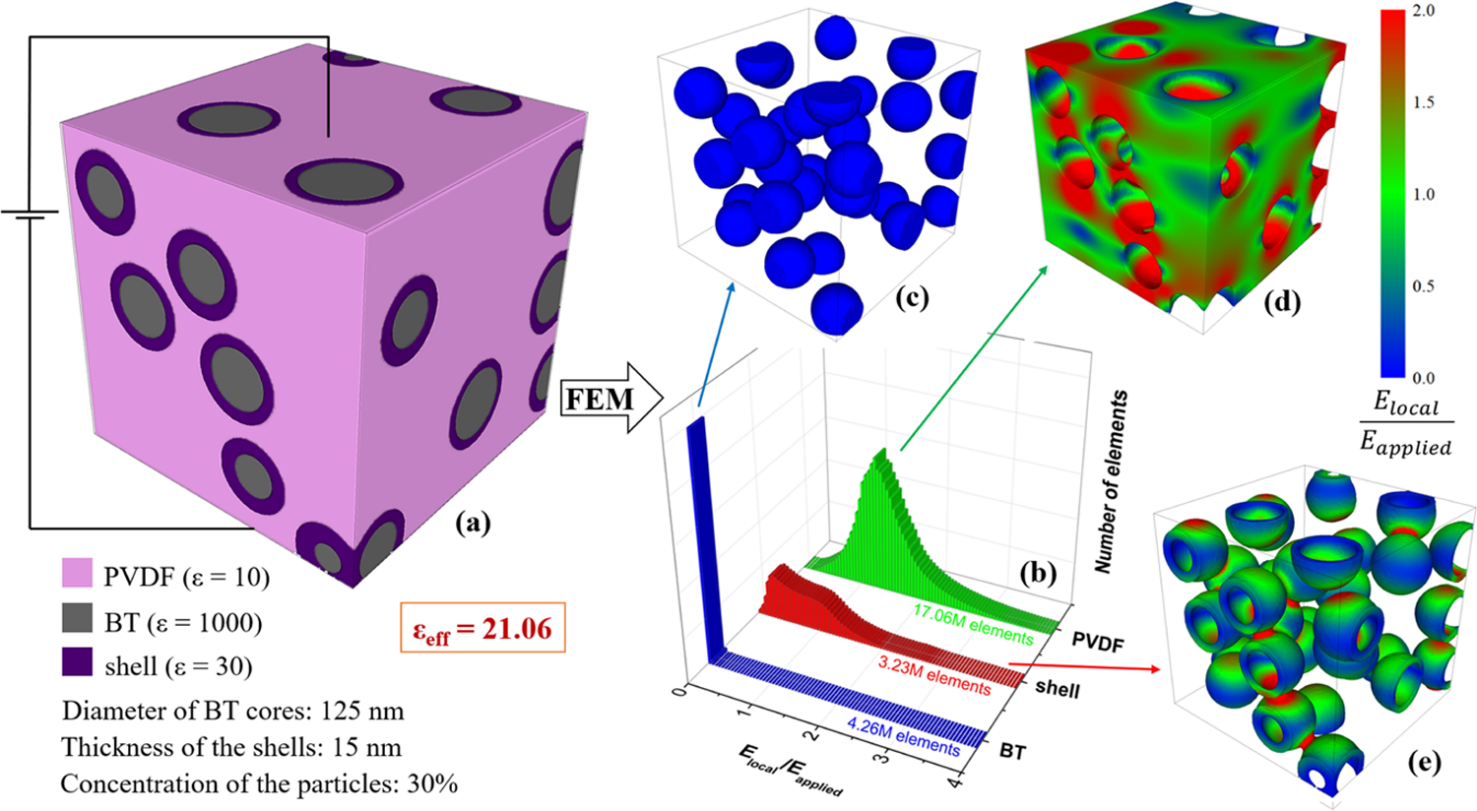
(a) Microstructure of a PVDF composite with BaTiO_3_@TiO_2_ inclusions (30 vol %), (b) local field distribution expressed on a number of elements basis and the local field maps in (c) the BT inclusions, (d) polymer matrix, and (e) titania shell, simulated by FEM. Shell thickness is 15 nm.

The effect of TiO_2_ coating is summarized in Figure 8 for a shell thickness of 15 nm. Again, the coating leads to zeroing the electric field in the BT phase, but now, the local field is enhanced on both the shell and matrix and is strongly inhomogeneous. In particular, the field on the titania phase is maximized at the contact points. The value of ε_eff_ is 20.4 for isolated inclusions and 21.06 for touching particles, while the experimental result is 19 for a shell thickness of 13 nm. Similarly to the effect of the SiO_2_ shell, a titania coating protects both the BaTiO_3_ particles and the PVDF matrix from breakdown^28,29^ with the advantage of a higher effective permittivity, which is twice that of the neat polymer. Consequently, a gain of a factor of about 2 for the discharged energy density can be expected for a loss-free composite in comparison to PVDF for the same applied field. The BDS of rutile is 800 kV/cm,^42^ but no data are available for amorphous TiO_2_. Composites containing 30 vol % of BaTiO_3_@TiO_2_ particles have a BDS of 3700–3800 kV/cm^28,29^ comparable with that of composites with BaTiO_3_@SiO_2_ inclusions,^20^ and this supports a rather high breakdown voltage also for amorphous titania.

The different behavior of the silica-coated and titania-coated inclusions is mainly determined by the dielectric constant of the shell. The dielectric constant of SiO_2_ is less than the permittivity of both PVDF and BaTiO_3_. Consequently, only the shell experiences high field values much larger than the applied field.

The above results identify 3D FEM simulations as a valuable tool for the design of dielectric composites with better properties. The calculation presented here can be extended to filler particles with different shapes and sizes and to explore architectures with improved dielectric response such as inclusions decorated with smaller particles of high permittivity, inclusions with multiple shells, star-like particles (and more generally particles with many spikes), and particles with high aspect ratio with embedded high-permittivity inclusions. In particular, fibers and sheets containing dispersed BaTiO_3_ nanoparticles have shown enhanced dielectric properties and BDS;^21,23,43^ similar architectures would deserve further FEM investigations. However, comparison of the FEM simulations with experimental results remains a fundamental step to gain further insight into processing–microstructure–property relationships of composite materials.

## Conclusions

In this paper, we have combined experimental data and 3D FEM simulations to shed some light on the role of microstructure on the dielectric properties of PVDF-BaTiO_3_ composites, with special attention to the effect of a thin shell of an oxide such as SiO_2_ or TiO_2_ on the perovskite surface. The main conclusions are as follows:

- In ideal composites with randomly distributed and well-isolated high-permittivity particles, the local field on the inclusions is virtually zero, while a strongly inhomogeneous and locally enhanced field (*E*_local_ > *E*_applied_) acts on the matrix. This is a consequence of the much higher permittivity of the inclusions (ε_r_′ ≈ 10^3^) in comparison to the polymer (ε_r_′ = 10). The increase of the effective permittivity with respect to the neat polymer value is only determined by the enhancement of the local field in the PVDF matrix and not by the contribution of the high-permittivity component to the total energy of the system.
- The presence of particle agglomerates and touching particles results in an increase of the local field in the contact region of the high-permittivity inclusions. *E*_local_ is highly dependent on the orientation of the contact point with respect to the applied field and is even larger than *E*_applied_. Therefore, ε_eff_ increases in comparison to the equivalent composite containing isolated particles owing to the additional contribution to the total electrostatic energy of the system determined by the enhancement of the field in the high-permittivity phase. However, the local field at the contact points can exceed the relatively low BDS (≈100 kV/cm) of the high-permittivity ferroelectric phase determining a deterioration of the breakdown strength of the composite.
- The use of core–shell inclusions provides an effective approach to overcome the degradation of the BDS in composites containing particle agglomerates because the local field in the contact region of the high-permittivity phase is again reduced to zero. The same consideration applies to percolating composites. When the permittivity of the shell is less than that of the polymer (the case of SiO_2_), the field in the matrix will be close to the applied field. Thus, the BDS of the composite will be largely determined by the breakdown resistance of the shell, whose composition should be carefully selected to sustain local fields several times larger than the applied field. When the permittivity of the shell is intermediate between that of the matrix and that of the core (the case of TiO_2_), an inhomogeneous distribution of the field with local enhancements will again take place in the matrix, but the field in the core is still zeroed. Even for isolated particles, the use of a shell with low permittivity will enhance the BDS by lowering the local field in the matrix. Core–shell inclusions represent an effective approach to resolve the paradox that composites with a high dielectric constant usually suffer from the low breakdown strength of the high-permittivity ferroelectric fillers, high dielectric losses, and high leakage currents. The main disadvantage is represented by the drop of permittivity, which decreases with increasing shell thickness. The use of very thin coatings can limit this problem.
- The above considerations apply to high-quality composites without porosity and good adhesion of the polymer matrix to the filler particles. Functionalization of the inclusions can be an effective solution to increase the polymer–filler compatibility. The contribution of additional dielectric relaxation phenomena associated with interfacial polarization or determined by a specific polymer coating can increase the effective permittivity of composites beyond the values predicted by the present FEM models.
- The 3D FEM simulations represent a valuable tool for the design of dielectric composites with better properties.

## Methods

### Preparation of Filler Particles

A commercial poly(vinylidene fluoride) (PVDF) grade provided by Solvay (Solef 1008) was employed as a polymer matrix for the preparation of the composites. In the following, some of its relevant characteristics are itemized: *M*_n_ = 114 × 10^3^ g/mol and *M*_w_ = 244 × 10^3^ g/mol, MFI@230 °C = 24 g/10 min (ASTM D1238, 5 kg), melting point 172 °C.^44^

BaTiO_3_ (BT) nanoparticles with an average diameter of 125 nm were synthesized with a hydrothermal-like method using BaCl_2_·2H_2_O (Aldrich, 99.9%) and TiCl_4_ (Aldrich, 99.9%) precursors as described in.^45^ The BT particles were used as such or coated with a silica (SiO_2_) or titania (TiO_2_) shell. BT@TiO_2_ inclusions were prepared by a two-step method previously used for coating different kinds of inorganic particles.^46^ First, the BT particles were suspended in a peroxotitanium(IV) solution at pH 9 prepared from TiCl_4_ (Aldrich, 99.9%). Subsequently, the hydrolysis of the peroxotitanium(IV) complex induced by heating the solution for 4 h at 95 °C determined the formation of an amorphous titania coating on the BT particles. The thickness of the shell was controlled by the titanium concentration in the suspension. The obtained titania-coated particles are denoted as BT-T*y* in the following, where T stands for TiO_2_ and *y* is the shell thickness (in nm).

The silica-coated particles (BT@SiO_2_) were prepared according to the modified Stöber-like process described by Mornet et al.^47^ In a typical synthesis, 0.5 g of BaTiO_3_ was dispersed by ultrasonication in 10 mL of nitric acid solution (1 M) and then in citric acid solution (0.01 M) and then washed with distilled water. Afterward, 200 μL of an ammonia solution (25 wt %) was added to a particle dispersion in distilled water. The obtained suspension was poured in a mixture of H_2_O/EtOH/NH_3_ (75/23.5/1.5 vol %) and reacted with a proper amount of tetraethyl orthosilicate (TEOS) solution (0.1 M in ethanol), added by dripping with a Hamilton syringe pump. The thickness of the shell was controlled by the amount of added solution.

The obtained silica-coated particles are denoted as BT-S*y* in the following, where S stands for SiO_2_ and *y* is the shell thickness (in nm). In both cases, the average thickness of the shell was determined from TEM images of the coated particles.

### Preparation of Composites

Composites containing 30 vol % of BT, BT@TiO_2_, and BT@SiO_2_ particles were prepared by solvent casting. The particles were dispersed in dimethylacetamide (DMA) by overnight mixing using YSZ media, and the final suspension was added to a solution of PVDF in DMA (20 wt %). The mixture was stirred at 90 °C until evaporation of most of the solvent and then poured on a glass substrate. The resultant film was finally dried in a vacuum and then in a vented oven; complete DMA removal was verified by attenuated total reflectance (ATR)-Fourier transform infrared (FTIR) spectroscopy. The obtained composites were compression-molded using a semiautomatic press P200E (Collin GmbH) using a two-step process previously optimized to obtain a high amount of ferroelectric β PVDF polymorph.^36^ In this procedure, a 3 mm thick plate of the composite is prepared at 200 °C and 50 bar for 4 min and then repressed at 170 °C and 240 bar for 4 min to obtain final sheets 0.6–0.8 mm thick. The composites were denoted with the same name of the corresponding filler particles inserting the prefix “C-.”

### Microstructural and Dielectric Characterization

Morphological analyses of the particles and the fragile fractured composite surfaces were carried out using a Hitachi TM3000 benchtop SEM microscope (15 kV) and, for higher magnification, an LEO 1450VP (LEO Electron Microscopy Ltd.) SEM microscope (20 kV). A Zeiss EM900 TEM microscope (80 kV) was also employed to study the inclusions morphology and determine the thickness of the binary oxide shell.

A FTIR spectrometer (PerkinElmer Spectrum Two) operating in ATR mode was used to characterize neat PVDF, ceramic particles, and their composites. The quantitative evaluation of the relative fraction, *F*_EA_, of the crystalline electroactive phase (β and/or γ) in the PVDF matrix was carried out as previously described, using the infrared spectra.^36^ The crystallinity degree (*X*_c_) of neat PVDF and of the composite materials was estimated with differential scanning calorimetry (DSC) using a Mettler DSC 821^e^ instrument and considering the method reported in ref (36).

For dielectric characterization, the sample surfaces were sputtered with Au–Pd to obtain circular electrodes (diameter: 2.4 cm), and frequency sweep measurements (1 Hz to 1 MHz) at room temperature were performed using a Solartron SI1260 frequency response analyzer with a Novocontrol BDC dielectric interface. The relative dielectric constant (real part of permittivity, ε_r_′) and tan δ (ratio of imaginary to real part of permittivity, ε_r_″/ε_r_′) were recorded.

Polarization vs field *P*(*E*) measurements were carried out at room temperature on electrode samples immersed in a transformer oil bath by a Sawyer–Tower modified circuit fed by a high-voltage sinewave (frequency: 10 Hz, maximum field amplitude: 460 kV/cm) using a TREK amplifier.

### Finite Element Modeling

The composites were simulated by a 3D approach, assuming a random distribution of spherical particles (30 vol %) in a polymer matrix. Three types of spherical inclusions were considered: neat BaTiO_3_, BaTiO_3_@SiO_2_, and BaTiO_3_@TiO_2_. The diameter of the BaTiO_3_ particles was 125 nm (corresponding to the average size experimentally determined), and the thickness of the shell was varied between 5 and 20 nm, corresponding to a shell volume fraction from 20.6 to 56.8%.

According to our previous experimental data^36^ recorded on polymer films processed with the same procedure, a value ε_r_′(PVDF) = 10 was assumed as representative of the relative dielectric constant of the PVDF matrix. The dielectric constant of the BaTiO_3_ particles was considered equal to 1000, a reasonable value for particles of this size, in agreement with recent measurements, ε_r_′ = 1150, for 100 nm particles.^48^ Indeed, the permittivity of barium titanate is strongly dependent on size,^49^ but considering that ε_r_′(BaTiO_3_) is much larger than that of the other phases, the exact value of the dielectric constant of barium titanate is practically irrelevant and determines only minor variations of the composite permittivity. Indeed, the BaTiO_3_ particles are subjected to very low local field values, and the increase of the effective permittivity is predominantly caused by the enhancement of the local fields located on the lower permittivity phases. A relative dielectric constant ε_r_′(SiO_2_) = 4 was assumed for silica, in agreement with the literature data of fused silica and silica-rich glasses.^42^ Although polycrystalline rutile TiO_2_ has ε_r_′ ≈ 100,^42^ amorphous titania has a lower permittivity, and values between 15 and 50 have been reported for thin films grown in different conditions.^50−53^ In the present calculations, it was assumed ε_r_′(TiO_2_) = 30. To avoid any confusion, the dielectric constant of the composites, as obtained from FEM calculations, is denoted by ε_eff_.

The microstructures of the composites (i.e., the way the spherical particles are arranged in the polymer matrix) have been randomly generated by considering a minimum distance of 130 or 120 nm between the inclusions’ centers to obtain either perfectly isolated particles or partially clustered inclusions, respectively. Cubic volumes of 1 μm^3^ having various types of fillers, as described before, were numerically generated and further divided into 27 million meshing elements. Further, the Laplace equation was solved using a Galerkin procedure described elsewhere,^54^ considering boundary conditions in a plan parallel-plate capacitor configuration with electrodes located on the top and bottom surfaces, subjected to a voltage of 1 V. This numerical procedure allows the calculation of the local potentials and electric fields and the determination of the effective permittivity from the total energy of the whole capacitor computed as a sum of electrostatic energy values of all of the discrete elements.

## Supporting Information Available

X-ray diffraction patterns of the BaTiO_3_ and BaTiO_3_@TiO_2_ powders and ATR-FTIR spectra of the neat PVDF and the composite films (PDF)
