# Supplementary material for: Role of Microstructures in the Dielectric Properties of PVDF-Based Nanocomposites Containing High-Permittivity Fillers for Energy Storage
Source: ACS Appl Mater Interfaces. 2023 Mar 2;15(10):13535–44. doi: 10.1021/acsami.2c23013 (PMC10020966; doi:10.1021/acsami.2c23013)
Supplement: Supplementary file 1 — am2c23013_si_001.pdf [file am2c23013_si_001.pdf]

Supporting Information

The role of microstructure on the dielectric properties of PVDF-based nanocomposites for energy storage containing high-permittivity fillers

Leontin Padurariu[a], Elisabetta Brunengo[b,c], Giovanna Canu[d], Lavinia Petronela Curecheriu[a], Lucia Conzatti[c], Maria Teresa Buscaglia[d], Paola Stagnaro[c], Liliana Mitoseriu[a], Vincenzo Buscaglia[d,*]

[a] *Faculty of Physics, Alexandru Ioan Cuza University, Blv. Carol I, nr.11, 700506 Iasi, Romania*

[b] *Department of Chemistry and Industrial Chemistry, University of Genoa, Via Dodecaneso 31, 16146 Genoa, Italy*

[c] *CNR-SCITEC, Institute of Chemical Sciences and Technologies "Giulio Natta", National Research Council, Via de Marini 6, 16149 Genoa, Italy*

[d] *CNR-ICMATE, Institute of Condensed Matter Chemistry and Technologies for Energy, National Research Council, Via de Marini 6, 16149 Genoa, Italy*

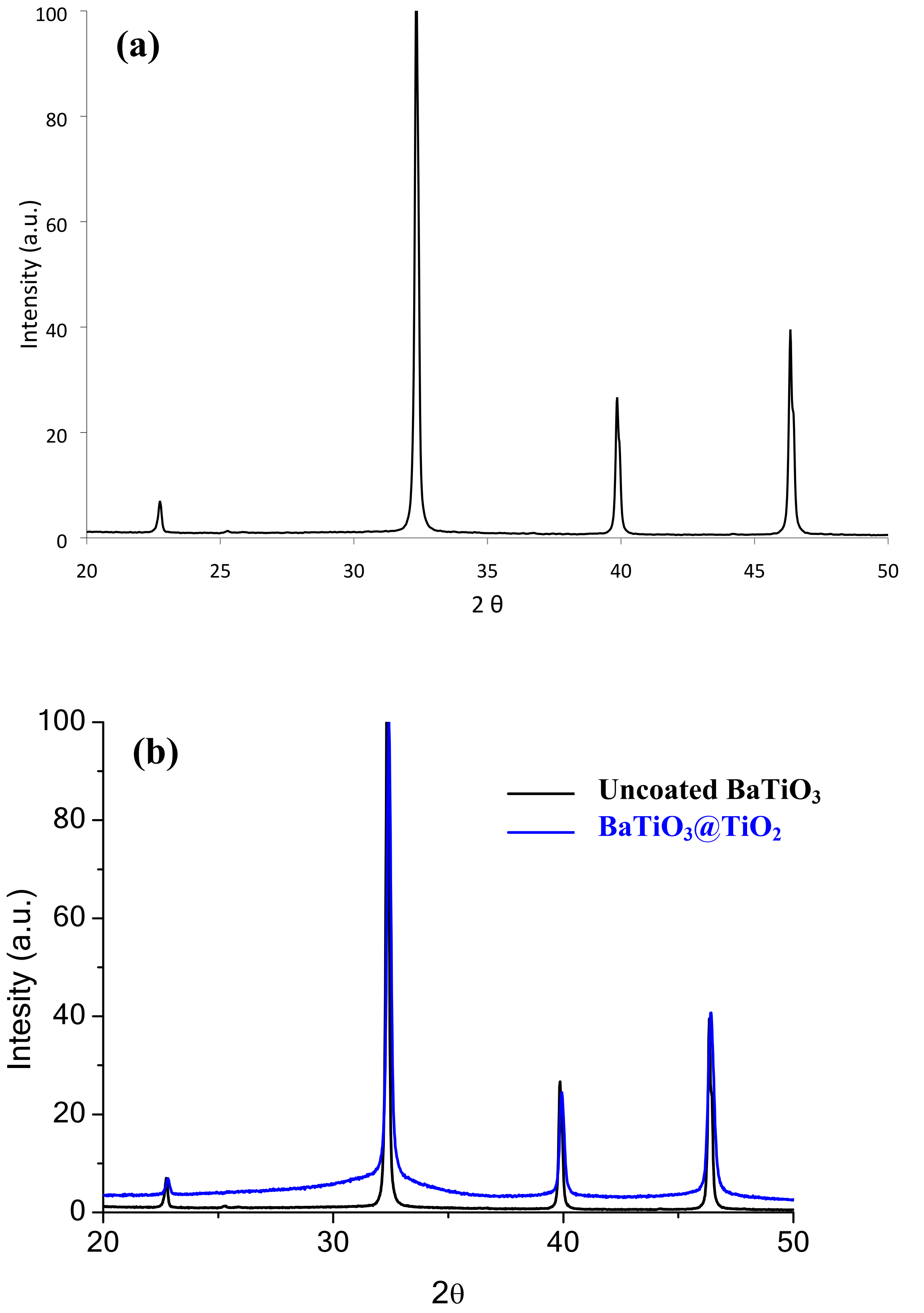

Figure S1. X-ray patterns of (a) uncoated $BaTiO_3$ powders and (b) comparison between uncoated $BaTiO_3$ powders (black) and $BaTiO_3$@TiO_2 powders (blue), showing the contribution of the amorphous titania coating.

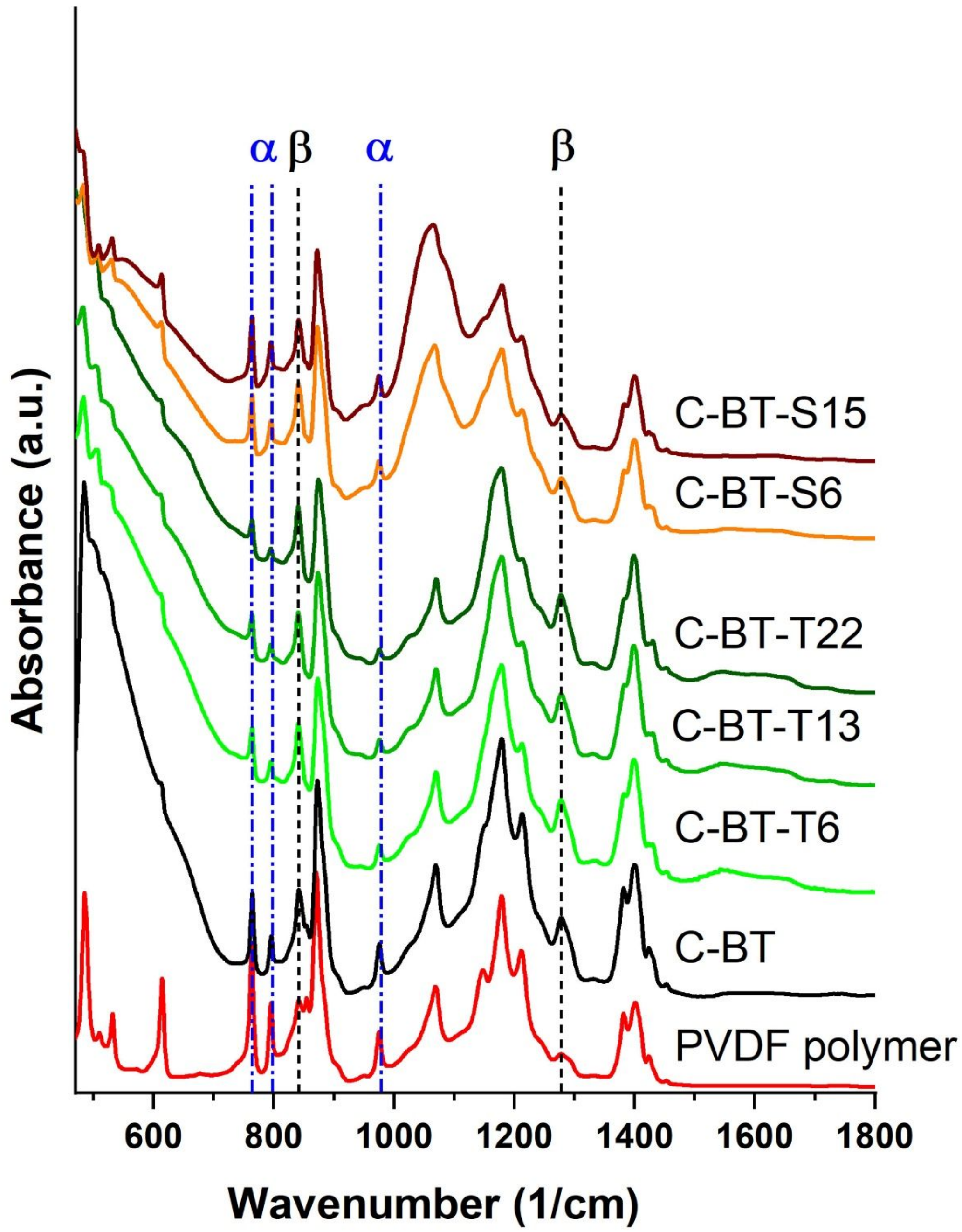

Figure S2. ATR-FTIR spectra of the neat PVDF polymer and PVDF-based composite films containing $BaTiO_3$ uncoated powders, $BaTiO_3@AO_2$, A = Ti, Si powders with different coating thickness as a filler
